# Supplementary material for: An infodemiologic review of internet resources on dental hypersensitivity: A quality and readability assessment
Source: PLoS One. 2025 Jan 24;20(1):e0312832. doi: 10.1371/journal.pone.0312832 (PMC11760580; doi:10.1371/journal.pone.0312832)
Supplement: S1 Appendix — (DOCX) [file pone.0312832.s001.docx]

Appendix 1: list of the included websites

| https://www.dentalhealth.org/sensitive-teeth |
| --- |
| https://www.rifkindental.com/blog/8-potential-causes-of-tooth-sensitivity |
| https://www.healthhub.sg/a-z/diseases-and-conditions/489/tooth_sensitivity_sdhf |
| https://healthify.nz/health-a-z/t/teeth-sensitive/ |
| https://www.australiadental.com.au/6-common-signs-sensitive-teeth/ |
| https://www.listerine-me.com/tooth-sensitivity/5-ways-soothe-sensitive-teeth |
| https://www.smilearizonadentistry.com/blog/home-remedies-sensitive-teeth |
| https://www.oda.ca/oral-health-basics/oral-conditions-diseases/tooth-sensitivity/ |
| https://truedental.ca/how-to-treat-sudden-tooth-sensitivity/ |
| https://www.coatbridgedentist.co.uk/suffering-from-sensitive-teeth-heres-how-to-relieve-your-pain/ |
| https://www.standrewsdentist.co.uk/sensitive-teeth |
| https://www.healthpartners.com/blog/why-are-my-teeth-sensitive/ |
| https://mypenndentist.org/dental-tips/2023/05/17/why-do-all-my-teeth-hurt/ |
| https://www.aestheticdentalbismarck.com/blog/tooth-sensitivity/ |
| https://www.sensodyne-me.com/en_AE/faqs.html |
| https://shelbourneclinic.ie/advice/sensitive-teeth/ |
| https://www.cccrdentistry.com/conditions/sensitive-teeth/ |
| https://www.wesleydentistry.com/10-tips-for-dealing-with- |
| https://www.arcdentalhealth.com/why-is-your-tooth-sensitive-to-col |
| https://www.medicoverhospitals.in/symptoms/tooth-sensitivity |
| https://www.oral-b.co.in/en-in/oral-b-institute/bad-breath/sensitive-teeth-complete-guide |
| https://www.woodhilldentalspecialties.com/help-my-teeth-hurt-5-reasons-why-theyre-sensiti |
| https://www.meyerwooddentistry.com/services/general-dentist/general-dentistry/sensitive-tooth-pain |
| https://www.charlotterootcanalcenter.com/blog/sudden-tooth-sensitivity |
| https://supremiadentistry.com/what-causes-sensitive-teeth-how-to-tr |
| https://grangefamilydental.com.au/why-are-my-teeth-sensitive/ |
| https://thelandingdentalspa.com/sensitive-teeth-causes-treatment-and-prevention/ |
| https://www.batemandentistry.com/blog/posts/guide-to-sensitive-teeth- |
| https://www.bostondentalgroup.com/blog/ouch-my-teeth-are-so-sensitive-6-possible-reasons-why/ |
| https://www.myidealdental.com/blog/why-are-my-teeth-sensitive-all-of-a-sudden/ |
| https://www.richmonddentalsuite.co.uk/factfiles/sensitive-teeth/ |
| https://www.andrewthomasdental.co.uk/help-common-causes-tooth-sensitiv |
| https://www.dentalcare.com/en-us/patient-education/english-articles/what-causes-sensitive-teeth |
| https://applecrossdental.com/faqs/what-causes-sensitive-teeth |
| https://www.mountlawleydental.com.au/blog/why-do-i-have-sensitive-teeth |
| http://www.dentistrathmines.com/sensitive-teeth.html |
| https://dentalhealthsociety.com/general/what-to-do-about-sensitive-teeth-and-when-to-tell-your-dentist/ |
| https://www.daasdentistry.com/your-basic-guide-to-the-causes-and-treatments-of-sensitive-teeth |
| https://www.dentalfearcentral.org/faq/sensitive-teeth/ |
| https://www.listerine-me.com/tooth-sensitivity |
| https://www.coatbridgedentist.co.uk/suffering-from-sensitive-teeth-here |
| https://thetoothbrushexpert.com/sensitive-teeth/ |
| https://www.elegantdentcare.com/blog/how-to-stop-sensitive-teeth-pain-immediately/ |
| https://www.nsdentist.com/blog/youre-hungry-but-youre-in-pain-now-what/ |
| https://www.listerine-me.com/tooth-sensitivity/frequently-ask-questions |
| https://kidodent.org/oral-and-dental-conditions/best-toothpaste-for-sensitive-teeth-fluoride-whitening-natural-and-more/ |
| https://www.medanta.org/patient-education-blog/home-remedies-for-sensitive-teeth |
| https://kidsdentalvisioncare.com/articles/kids-sensitive-teeth-causes-symptoms-and-treatment |
| https://www.oralb.co.uk/en-gb/oral-health/conditions/teeth-sensitivity/sensitive-teeth-causes-treatments-prevention |
| https://oralhealthcomplete.com/how-to-stop-sensitive-teeth-pain-immediately-7-treatments-remedies/ |
| https://www.interdent.com/gentle-dental/resources/teeth-sensitivity-causes/ |
| https://www.wesleydentistry.com/10-tips-for-dealing-with-sensitive-teeth/ |
| https://www.mayoclinic.org/healthy-lifestyle/adult-health/expert-answers/sensitive-teeth/faq-20057854#:~:text=Sensitive%20teeth%20are%20typically%20the,worn%20filling%2C%20or%20gum%20disease. |
| https://westclairdental.com/what-are-the-symptoms-of-tooth-sensitivity/ |
| https://www.topdoctors.co.uk/medical-dictionary/tooth-sensitivity |
| https://www.bostondentalgroup.com/blog/dental-hypersensitivity-what-it-is-and-how-to-treat-it/ |
| https://snodgrassking.com/treating-tooth-sensitivity/ |
| https://www.sensodyneca.com/en_CAM/acerca-de-la-sensibilidad/causas-de-la-sensibilidad-dental.html |
| https://www.yourdentistryguide.com/tooth-sensitivity/ |
| https://www.vinmec.com/en/news/health-news/what-are-sensitive-teeth-what-is-the-cause/ |
| https://www.philips.com.au/c-m-pe/dental-professionals/dental-indications/tooth-sensitivity |
| https://www.soldentalcare.com/dental-blog/tooth-sensitivity-to-cold-and-sweets/ |
| https://www.arcdentalhealth.com/why-is-your-tooth-sensitive-to-cold/ |
| https://www.citybridgedental.co.uk/articles/4-highly-effective-treatments-for-sensitive-teeth |
| https://www.chelmsforddental.com.au/tooth-sensitivity/ |
| https://www.sakraworldhospital.com/blogs/suffering-from-tooth-sensitivity-heres-what-you-need-to-know/225 |
| https://www.emergencydentistsydney.com.au/news/dental-hypersensitivity |
| https://www.beacondentalhealth.com/blog/why-are-my-teeth-sensitive-all-of-a-sudden/ |
| https://www.premierdentalohio.com/blog/how-to-deal-with-tooth-sensitivity |
| http://www.drmarclazare.com/blogs/2020/september/tooth-sensitivity-cause-treatments/ |
| https://yotuel.com/en/blog/dental-hypersensitivity-what-is-it-and-how-to-alleviate-it/ |
| https://www.cheadlehulmedental.com/dentin-hypersensitivity-what-is-it-and-what-helps/ |
| https://greensboro-dentist.com/what-causes-sensitive-teeth/ |
| https://nollfamilydentistry.com/blog/tooth-sensitivity/ |
| https://kidshealthyteeth.com/stop-the-pain-how-you-can-relieve-sensitive-teeth/ |
| https://www.vdsclinic.com/what-is-teeth-hypersensitivity-and-how-does-it-develop-after-tooth-fillings-or-bondings/ |
| https://www.dentaid.com/en/oral-health/dental-sensitivity |
| https://www.hinsdaledental.com/blog/what-causes-tooth-sensitivity-how-to-treat-it/ |
| https://www.carebywally.com/blog/tooth-sensitive-to-pressure |
| https://www.choice-dental.com.au/types-of-tooth-sensitivity-symptoms/ |
| https://www.capronparkdental.com/teeth-sensitivity.html |
| https://www.perkinsdentalbaltimore.com/blog/what-causes-tooth-sensitivity/ |
| https://www.thetotaldentistry.com/2020-03-hypersensitivity-of-teeth-what-is-it-and-how-to-deal-with-it/ |
| https://www.usa.philips.com/c-m-pe/dental-professionals/dental_indications/tooth-sensitivity |
| https://dentalsolutionsclinic.com/treatments/desensitisation-dental-laser/ |
| https://y-brush.com/en/blogs/y-brush/what-is-tooth-sensitivity-and-how-to-care-for-sensitive-teeth |
| https://sierradental.ca/teeth-sensitive-dental-cleaning/ |
| https://www.belmarorthodontics.com/tooth-sensitivity-with-braces/ |
| https://healthcare.utah.edu/healthfeed/2017/12/perils-of-sensitive-teeth |
| https://www.colgate.com/en-us/oral-health/tooth-sensitivity/potassium-nitrate-dental-uses-relieving-your-tooth-sensitivity |
|  |
