# Supplementary material for: An infodemiologic review of internet resources on dental hypersensitivity: A quality and readability assessment
Source: PLoS One. 2025 Jan 24;20(1):e0312832. doi: 10.1371/journal.pone.0312832 (PMC11760580; doi:10.1371/journal.pone.0312832)
Supplement: Attachment — Submitted filename: PLOS Peer Review.docx [file pone.0312832.s003.docx]

Investigating how patients seek and incorporate information about oral health is vitally important for understanding the questions they ask dental professionals and the types of treatments they prefer. You have done a tremendous amount of work on this study, and I appreciate the time and care you spent on the analysis.

I do, however, have a few questions about this manuscript.

In the first paragraph of the Introduction, you cite a paper from the WHO which states 71% of internet users seek health-related information. A few pages later, you cite a paper by Cline and Haynes which states that there are over 70,000 websites that provide health related content that are visited by more than 50 million users. These numbers seemed a bit off to me, and a quick check of the References showed that these papers are from 2007 and 2001 respectively. When reporting on the information seeking habits of internet users staying completely current is difficult as tools literally change daily, but there must be better sources of data than these. A quick check of Google Scholar (which admittedly is not without its own concerns) shows that Cline and Haynes have been cited more than 2,300 times with more than 500 of these citations coming in the last five years. Perhaps one of these papers would have more recent data that could be used instead.

Too much of the Introduction is also devoted to the various causes of hypersensitivity and tooth pain. This would be relevant if patients were searching for specific causes of their discomfort (such as something like “tooth sensitivity cold” or “tooth sensitivity hard toothbrush”), but the searches documented only show "Sensitive teeth" and "Teeth hypersensitivity" which are much broader.

I also have a question about this question itself; you write, “In the field of dentistry, patients often seek health information regarding teeth hypersensitivity. It is common for patients to turn to the internet for information regarding dental health issues such as tooth hypersensitivity”. Is this anecdotal? Is this a topic patients often ask about in the office? I did a quick search in Google Trends for the broader term you used (Tooth Sensitivity) and it didn’t register as a trending topic. I broadened this out to the last year and compared it to what Google reported as a more common related search (Tooth Pain) and here are the results:

I don’t have access to more powerful analytics platforms like Semrush or Similarweb, but I did a similar search using Wikipedia’s Pageviews Analysis for Tooth Sensitivity and Toothache (which is the parent term for Tooth Sensitivity on the platform):

I have no doubt that Tooth Sensitivity is of supreme interest to patients suffering from its effects, but it’s not as common as other concerns to internet users. Was this topic chosen to make analyzing the results more manageable? If so this is fine, real world concerns like this are valid, but this should be noted if this indeed a conscious choice.

In the Methods and Materials you mention that you used Google Chrome in Incognito mode to minimize impact from cookies and to eliminate bias. As you may have read, Google recently settled a class-action lawsuit in the United States due to years of misleading claims about the degree of anonymity provided by incognito mode. I realize that this information was made public after this manuscript was prepared and after your study was conducted, but the objectivity of Google’s SEO has always been suspect. I appreciate the steps you took to try to make these searches as transparent and reproducible as possible, but using a search engine like DuckDuckGo or a browser like Tor would be a better option.

While thinking about the Methods, how did you decide upon analyzing a total of 600 websites? With over 30,000,000 results choosing a Representative Sample (say even 1%) would be daunting, but there needs to be some explanation as to how this choice was made. Did you consult with a statistician? Did you follow the approach used by a similar study? Did you try to mimic the behavior of the average searcher? If this is the case 100 results from each search is far too large as previous studies have noted that the vast majority of users rarely click through more than the second page of results, so perhaps the first 20 would be a more common sample size. It’s also true that users can rarely access more than the first 1,000 results from a search engine, so creating a true Representative Sample is difficult without access to advanced tools and metrics.

If you were to employ Systematic Sampling you could use each search engine’s Advanced Search features to categorize the results. For example, in Google’s Advanced Search you could limit by Site or Domain. You stratified the websites selected for analysis by dental or medical centers, commercial entities, non-profits and governments or universities. You could do this in Google by choosing domains like .com, .org, .gov and .edu. This would allow you to choose the top 100 (or whatever number you choose) websites from each domain. This may lead to a fairer comparison of the readability and quality of offerings from each type of entity.
