# Supplementary material for: An infodemiologic review of internet resources on dental hypersensitivity: A quality and readability assessment
Source: PLoS One. 2025 Jan 24;20(1):e0312832. doi: 10.1371/journal.pone.0312832 (PMC11760580; doi:10.1371/journal.pone.0312832)
Supplement: Attachment — Submitted filename: Respond to reviewers.docx [file pone.0312832.s004.docx]

Respond to reviewers

Comment: Minor error in Figure 2 - "Fair Eaesy" is mispelled. Will need to be corrected in the software that produced the figure.

Response: corrected
