# Supplementary material for: An infodemiologic review of internet resources on dental hypersensitivity: A quality and readability assessment
Source: PLoS One. 2025 Jan 24;20(1):e0312832. doi: 10.1371/journal.pone.0312832 (PMC11760580; doi:10.1371/journal.pone.0312832)
Supplement: Attachment — Submitted filename: Response to reviweres .docx [file pone.0312832.s005.docx]

Dear respected editor and reviewers,

We would like to extend our thanks to the editor and the respected reviewers for the valuable and constructive comments. We confirm that we have gone over all comments, responded to all comments/suggestions and done all required corrections, as outlined below. All corrections/additions in the text have been marked in blue.

**Journal Requirements:**

Please review your reference list to ensure that it is complete and correct. If you have cited papers that have been retracted, please include the rationale for doing so in the manuscript text, or remove these references and replace them with relevant current references. Any changes to the reference list should be mentioned in the rebuttal letter that accompanies your revised manuscript. If you need to cite a retracted article, indicate the article's retracted status in the References list and also include a citation and full reference for the retraction notice.

**Author’s Response:** Thank you for your comment. Upon careful review, we confirm that no changes were made to the reference list in the revised manuscript, and no retracted articles were cited. The reference list remains complete and correct as per the original submission.

**Additional Editor Comments:**

Thank you for revising the article. Considering the reviewers' comments, please address the mentioned issues and submit the revised version to the journal.

**Author’s Response:** Thank you for your feedback. We appreciate the opportunity to revise our article based on the reviewers' valuable comments. We have carefully addressed all the issues raised and made the necessary revisions in the manuscript. Thank you for your thoughtful feedback.

**Reviewer #4**: As demonstrated in the final manuscript, the authors have made a significant effort in applying the reviewers' corrections, and therefore, I believe this research deserves to be published.

**Author’s Response:** We truly appreciate your recognition of the effort put into addressing the reviewers' corrections. We are pleased that the revisions have met the expectations and standards of the review process, and we are grateful for your support in recommending this research for publication.

**Reviewer #5:** The topic is interesting. However, I would like to recommend the author to-

1. explain why the instruments were chosen to use in this study

**Author’s Response:** Thank you for your comment. We mentioned the important role of each tool in method section and based on your comment we added the following to discussion section “The HONcode, DISCERN, and JAMA Benchmarks are among the most widely utilized instruments for evaluating the reliability and quality of information presented on medical websites”

2. explain the technical terms specifically for those which are essential to the study such as the Kruskal-Wallis test, as the readers could more understanding.

**Author’s Response:** Thank you for your comment. In response to your feedback, we have amended the statistical analysis subsection to include the following: "The Kruskal-Wallis test is a non-parametric statistical test utilized to assess whether there are significant differences between the medians of three or more independent groups. This test serves as an alternative to the one-way ANOVA when the assumptions of normality are not satisfied."

3. specify in the meterial and method part regarding the websites that used to collect data and explain why they were chosen.

**Author’s Response:** Thank you for your comment. Part to explain as mentioned “Using Google Chrome, version 81.0.4044, we conducted searches on Yahoo! (http://www.yahoo.com), Bing (http://www.bing.com), and Google (http://www.google.com). This approach is in line with the findings of "The Pew Research Center's Internet and American Life Project" [2], which reports that 79% of individuals who search for health information online utilize these search engines"

4. In discussion, the keywords "dentin sensitivity, cervical hypersensitivity, cemental hypersensitivity, and root hypersensitivity" are different from the result in Meterial and Methods part.

**Author’s Response:** Thank you for directing our attention to this matter; we have amended the inconsistency.

5. For future study, it is recommend that the author should use the information sources that donot share the same index. So, this could be beneficial for the readers to choose the searche engine when they want more or different information since Bing and Yahoo share the same indexing.

**Author’s Response:** Thank you for this valuable suggestion we added a brief paragraph based on your suggestion to discussion. In future studies, diversifying information sources beyond those sharing the same index, such as Bing and Yahoo, can enhance the breadth of research findings. By incorporating varying search engines, readers gain access to a wider array of perspectives and data, allowing for more comprehensive and varied insights. This approach not only enriches the research but also aids readers in selecting search engines that better meet their informational needs.

6. It is strongly recommended that the significances-benefits or outcomes, of this study should be clearly highlighted in the conclusion.

**Author’s Response:** Thank you for this valuable suggestion we added a brief statement based on your suggestion to conclusion. “This study highlights the moderate quality and challenging readability of online health information on dental sensitivity. The findings underscore the need for improved reference standards and HONcode certification to enhance reliability. By identifying gaps in current online resources, this research emphasizes the importance of developing accessible and trustworthy health information for patients.”

**Reviewer #6:**

This paper is well written and easy to read.

Font size or a couple of citations in references look inconsistent. This also happens to a couple of pages in the body of text.

**Author’s Response:** Amended

**Reviewer #7**:

In general, the author has answered the reviewer(s) ' questions, which improves the quality of the manuscript in this version.

**Author’s Response:** Thank you for your positive feedback. We are glad to hear that the revisions have satisfactorily addressed the reviewers' questions and have contributed to improving the quality of the manuscript.

Some additional suggestions are listed below.

1. Author affiliation.

Changing an author's affiliation should be done according to the rules of the journal. In general, the unit that recognizes a researcher means that the research was completed during his tenure at that unit, not that it changed when he changed

jobs.

**Author’s Response:**

I would like to kindly inform you that the research presented in this manuscript was conducted during my tenure at my current affiliation, King Fahad Hospital. At the time of conducting this study, I was concurrently affiliated with Taibah University, but all the research work and contributions were completed while I was affiliated with King Fahad Hospital.

I believe it is appropriate to reflect my current affiliation in the publication, and I appreciate your understanding in this matter. Please let me know if you need any additional information or clarification.

2. Typos. The system shows that the author claims to use four search engines, but the article talks about three (i.e., Yahoo, Bing, and Google). Please correct this.

**Author’s Response:** Thank you for directing our attention to this matter; we have amended the inconsistency.

3. Limitation and direction.

The author(s) stated that they followed the literature guidelines regarding the three search engines used (Yahoo, Bing, and Google) and listed their limitations in terms of information coverage in the limitations section of the study.

In fact, due to Internet use regulations and other restrictions, some users in China may also use Baidu to search for English-language health information. Perhaps the researchers can list future research directions, such as comparing the results of search engines with different user bases.

Thank you for this valuable suggestion we added a brief paragraph based on your suggestion to discussion. “Future research could explore the effectiveness and coverage of other search engines like Baidu, especially in regions with different internet regulations. By comparing search engines with distinct user bases, such as Baidu, Yahoo, Bing, and Google, researchers can gain insights into how users access English-language health information across diverse geographic and cultural contexts. This could help identify gaps in information availability and inform strategies to improve online health information dissemination globally. Additionally, examining user behavior and preferences on these platforms could provide a deeper understanding of search engine optimization and its impact on health literacy.”
